# The Role of Cardiac Magnetic Resonance Imaging in Severe Anorexia Nervosa

**DOI:** 10.7759/cureus.4229

**Published:** 2019-03-11

**Authors:** Karen Chu, Chantal Y Asselin, Ilan Buffo, Margo Lane, Louis Ludwig, Davinder S Jassal, Daryl Schantz

**Affiliations:** 1 Internal Medicine, University of Manitoba, Winnipeg, CAN; 2 Cardiology, University of Manitoba, Winnipeg, CAN; 3 Pediatrics, University of Manitoba, Winnipeg, CAN; 4 Psychiatry, University of Manitoba, Winnipeg, CAN

**Keywords:** anorexia nervosa, cardiac magnetic resonance imaging, transthoracic echocardiography, cardiovascular remodelling

## Abstract

Objective

Anorexia nervosa (AN) patients are at an increased risk of developing cardiac complications including bradyarrhythmias, systolic dysfunction, pericardial effusions, and sudden cardiac death. Although previous echocardiographic studies in AN patients have demonstrated a reduction in overall left ventricular (LV) mass, systolic dysfunction, and silent pericardial effusions, little is known about the role of cardiac magnetic resonance imaging (CMR) in assessing this patient population. The objective of this study was to assess cardiac indices and the presence of myocardial fibrosis in AN patients.

Methods

Between 2014 and 2015, a cross-sectional pilot study of 16 female patients who met the Diagnostic and Statistics Manual of Mental Disorders, fifth edition (DSM-5) criteria for AN was conducted at a single tertiary care center. Baseline characteristics including age, weight, food restriction behavior, over-exercise, self-induced vomiting, and laxative abuse were collected in the study population. Electrocardiography, transthoracic echocardiography (TTE), and CMR were performed.

Results

The mean age was 17 years (range: 13-22 years). There were no conduction abnormalities as the average PR interval was 152 ms (range: 130-190 ms) and QTc was 413 ms (range: 360-450 ms). Using TTE, the left ventricular ejection fraction (LVEF) was 54 ± 4% with a lower LV mass/body surface area (BSA) of 56 ± 7g/m^2 ^in AN patients as compared to controls. Using CMR, both the mean LVEF of 52 ± 9% and LV mass/BSA of 45 ± 4g/m^2 ^were lower in AN patients as compared to controls. Using CMR, both right ventricular ejection fraction (RVEF) of 50 ± 10% and a right ventricular (RV) mass/BSA of 18 ± 3g/m^2 ^were smaller in AN patients as compared to controls. There was no evidence of late gadolinium enhancement (LGE) in the study population.

Conclusions

Young patients with AN have lower cardiac mass and volumes with no evidence of myocardial fibrosis.

## Introduction

Anorexia nervosa (AN) is a disease characterized by fear of weight gain, caloric restriction, and distorted self-image affecting 0.3% to 1.0% of Canadians [1]. Cardiac complications, including sinus bradycardia, orthostatic tachycardia, decreased myocardial mass and dysfunction, silent pericardial effusions, and an increased risk of sudden cardiac death (SCD), have been reported in up to 80% of patients [2-7]. As compared to other psychiatric illnesses, AN patients have an increased mortality rate [8].

Previous echocardiographic studies have demonstrated that AN is associated with a smaller left ventricular (LV) mass, reduced LV cavity volumes, and lower cardiac index (CI); various mechanisms have been described to explain these findings [4]. Previous studies have also demonstrated that in AN patients, the finding of a reduced LV mass is associated with a prolonged QT corrected (QTc) [3,5]. However, it is unclear if this finding increases rates of SCD in the AN population as reported cases of Torsade de Pointes are rare. Studies are contradictory on the effects of AN on LV systolic function [4]. Older studies demonstrated a reduced left ventricular ejection fraction (LVEF) in AN patients and recent studies demonstrate a normal LVEF in this patient population [6-7]. Additionally, silent pericardial effusions also occur in up to 70% of AN patients and resolve with re-feeding [4]. Despite these structural findings, transthoracic echocardiography (TTE) is limited because of its dependency on acoustic windows, poor apical and right ventricular (RV) visualization, and the inability to detect myocardial fibrosis.

Cardiac MRI (CMR) is able to overcome some of these limitations with increased spatial resolution. It is also able to non-invasively detect myocardial fibrosis, marked by the presence of late gadolinium enhancement (LGE) [9]. Prior studies comparing TTE and CMR have demonstrated improved LVEF and right ventricular ejection fraction (RVEF) quantification and detection of regional wall motion abnormalities using the latter technique [10]. To our knowledge, only one study reported the CMR findings in a group of adult AN patients [11]. The authors found a smaller LV mass compared with a control population and the presence of myocardial fibrosis in 23% of the study population [11]. However, their study did not evaluate whether there were changes to RV structure and function in their patient population.

The objective of this study was to determine the amount of change in cardiac indices and assess for myocardial fibrosis in AN patients using both TTE and CMR.

## Materials and methods

Study population

A prospective cross-sectional pilot study was performed on 16 adolescent/ young adult female patients diagnosed with severe AN who were followed up as outpatients by the Eating Disorders Service at a single tertiary care center. All patients were between the ages of 12 and 25 years, met the Diagnostic and Statistical Manual of Mental Disorders, fifth edition (DSM-5) diagnostic criteria for AN, and had a weight at diagnosis of less than 80% expected body weight in their convalescence phase. Exclusion criteria included poor renal function (serum creatinine >100 mmol/L), hemodynamic instability, or other contraindications to CMR. The control population included normal values obtained from published literature. The study protocol was reviewed and accepted by the local ethics board (REB number H2013:378).

Electrocardiogram

A standard 12-lead electrocardiogram (ECG) was performed for measurement of heart rate, PR interval, and QT interval. The QTc was calculated using Bazett’s formula [12].

Echocardiography

Standard TTE was performed using a Phillips iE33 machine (Philips, Andover, MA, USA) and a multi-frequency transducer [S8-3 (3-8 MHz) and X5-1 (1-5 MHz) probes]. Parasternal long axis (PLAX) views were used to determine left ventricular end-diastolic diameter (LVEDD), left ventricular end-systolic diameter (LVESD), posterior wall thickness (PWT), and interventricular septal thickness (IVS). The right ventricular diameter was measured at the base of the RV apical view. LVEF was calculated using Simpson’s method from the apical four-chamber view [13-14]. LV mass was calculated using LV linear dimensions as per the American Society of Echocardiography recommendations [13-14]. Valvular dysfunction was determined qualitatively. If the severity was deemed more than mild, regurgitant lesions were quantified using the proximal isovelocity surface area (PISA) method and stenotic lesions quantified using the continuity equation [15-16]. The presence of pericardial effusion was determined. All TTE studies were interpreted offline using Philips Xcelera (Andover, MA, USA) by a single observer (DS). 

Cardiac magnetic resonance imaging

All CMR scans were performed using a 1.5 Tesla Siemens Scanner (Sonata, Magnetom, Siemens, Erlangen, Germany) at a single tertiary care center. Segmented TrueFISP sequences were performed with ECG gating to obtain images representative of the entire cardiac cycle in both long and short axis views. Three- and four-chamber long axis views were used to measure chamber dimensions. Tracing the endocardial and epicardial borders manually on consecutive short axis slices of the LV and RV allowed quantification of the LVEF, LV mass, LV stroke volumes, RVEF, and RV mass. End-diastole and end-systole were determined visually using the slices with the largest and smallest ventricular volumes, respectively. Two blinded observers (KC and DS) analyzed the CMR imagines offline using a CMR analysis software (version 3.4.0, Circle Cardiovascular Imaging, Calgary, Alberta, Canada).

Statistical analysis

Continuous data points were reported as mean ± standard deviation (SD). Comparison between measurements in the AN population and control population (normal values in published literature) was done by the two-tailed one-sample t-test method. The published normal values to which we compared our patients included pediatric and adult patients who were also stratified by sex allowing us to compare patients to a population with similar age and sex [17]. A *p*-value <0.05 was considered statistically significant. Statistical analysis was performed using the GraphPad Prism 7 software (GraphPad Software, La Jolla, CA, USA).

## Results

Study population

The baseline characteristics of the study population (*n *= 16) are shown in Table 1. The average age at the time of diagnosis of AN was 13.1 ± 1.7 years with an average percentage expected body weight of 73 ± 4%. At the time of CMR, the average age was 17 ± 3 years, the percentage expected body weight was 88 ± 12%, height was 166 ± 13cm, body mass index (BMI) was 20 ± 3 kg/m^2^, and body surface area (BSA) was 1.6 ± 0.2 m^2 ^for the study population.

**Table 1:** Baseline characteristics of anorexia nervosa study population (n = 16) AN, anorexia nervosa; CMR, cardiovascular magnetic resonance imaging; BMI, body mass index; BSA, body surface area

At the time of diagnosis of AN	
Age (years)	13.1±1.7
% expected body weight	73±4
At the time of CMR:	
Age (years)	17±3
% expected body weight	88±12
BMI (kg/m^2^)	20±3
Height (cm)	166±13
BSA (m^2^)	1.6±0.2

Electrocardiography

The average heart rate was 65 ± 13 (45-90) bpm, average PR interval 149 ± 19 (120-190) ms, and the average QTc was 413 ± 22 (360-450) ms. There was no evidence of first-degree atrioventricular (AV) block nor a prolonged QTc. 

Left ventricular geometry and systolic function

The structural and functional parameters on TTE and CMR are shown in Tables 2, 3, respectively. As compared to controls, there was a statistically significantly lower LVEF by TTE (Table 2). Also, as compared to controls, there was a statistically significantly lower LV mass index in AN patients on TTE (56 ± 7 g/m^2 ^vs. 69 ± 26 g/m^2^, *p *< 0.05). Using CMR, AN patients had a statistically significantly lower LVEF, LV mass index, PWT, and CI as compared to controls (Table 3). There was no evidence of LGE in any of the study participants. A total of two patients (12.5%) had evidence of a small pericardial effusion. 

**Table 2:** Echocardiographic parameters in study population (n = 16) LVEF, left ventricular ejection fraction; RV, right ventricle; IVS, interventricular septal thickness; PWT, posterior wall thickness; LVEDD, left ventricular end diastolic diameter; LVESD, left ventricular end systolic diameter; AN, anorexia nervosa

	Control	AN patients (n = 16)	p-value
LVEF (%)	64 ± 5	54 ± 4	p < 0.05
RV diameter (mm)	27 ± 4	13±8	p < 0.05
LV IVS (cm)	6.0 ± 1.5	6.7 ± 1.2	NS
LV PWT (cm)	6.0 ± 1.5	6.0 ± 1.0	NS
LVEDD (cm)	45.0 ± 3.6	45.2 ± 3.4	NS
LVESD (cm)	28.2 ± 3.3	27.2 ± 2.4	NS
LV mass index (g/m^2)^	69 ± 26	55.5 ± 6.5	p < 0.05
Aortic sinus diameter (cm)	23 ± 2	24.5 ± 1.8	p < 0.05

**Table 3:** Cardiac magnetic resonance imaging parameters in the study population (n = 16) LVEF, left ventricular ejection fraction; RV, right ventricle; IVS, interventricular septal thickness; PWT, posterior wall thickness; LVEDD, left ventricular end diastolic diameter; LV SVI, left ventricular stroke volume index; CI, cardiac index; LA, left atrium; RVEDD, right ventricular end-diastolic diameter; RVEF, right ventricular ejection fraction; RV SVI, right ventricular stroke volume index; AN, anorexia nervosa

	Controls	AN patients (n = 16)	p-value
LVEF (%)	67 ± 5	52 ± 9	p < 0.05
LV IVS (mm)	11 ± 2	8.6 ± 4.1	p < 0.05
LV PWT (mm)	11 ± 1	8.4 ± 2.4	p < 0.05
LVEDD (mm)	45 ± 4	41.3 ± 3.5	p < 0.05
LV SVI (mL/m^2^)	52 ± 6	39 ± 3	p < 0.05
LV mass index (g/m^2^)	62 ± 8	45 ± 4	p < 0.05
CI (L/min/m^2^)	3.3 ± 0.4	2.8 ± 0.4	p < 0.05
LA diameter (3 chamber view)	31 ± 0.5	21.5 ± 5.1	p < 0.05
RVEDD (mm)	35 ± 5	21.4 ± 3.7	p < 0.05
RVEF (%)	64 ± 6	50 ± 10	p < 0.05
RV SVI	50 ± 6	36 ± 8	p < 0.05
RV mass index (g/m^2^)	30 ± 5	18 ± 3	p < 0.05

Right ventricular geometry and systolic function

The structural and functional parameters on TTE and CMR for the right ventricle are shown in Tables 2 and 3, respectively. As compared to controls, there is a statistically significantly lower RV diameter using TTE in the AN study population. Using CMR, the RV diameter, RVEF, RV stroke volume index, and RV mass index are statistically smaller in AN patients. 

## Discussion

Patients with AN may develop a number of cardiac complications [2-7]. These include ECG abnormalities (heart rate and repolarization abnormalities), pericardial changes, hemodynamic changes, and structural changes. 

ECG findings and repolarization changes

Our study documented a normal heart rate, PR interval, and QTc interval in adolescent and young adult girls with AN. QTc prolongation is noted in some reports of AN, but not noted in others [3,5,18-19]. When present, it tends to normalize after weight recovery [18]. Abnormal QTc values observed in these studies may be secondary to electrolyte abnormalities common in AN [3]. In all of our patients, the QTc was normal which is consistent with the study by Facchini et al. [19].

Pericardial changes

Previous echocardiographic studies have demonstrated a higher prevalence of pericardial effusions in AN patients. Docx et al. prospectively studied 128 female AN patients (9-18 years) demonstrating the presence of pericardial effusion in >20% of patients [20]. Most of the patients (66%) had a small pericardial effusion between 0.3-0.8 cm in dimension. The remainder had a moderate to large pericardial effusion (>0.8 cm). There was no clinical or echocardiographic evidence of tamponade physiology in any of the patients. Patients at the highest risk of developing a pericardial effusion were those with a BMI ≤13.5kg/m^2 ^or a weight loss of ≥25%. A separate study by Frölich et al. retrospectively reviewed 65 AN patients (15.2 ± 3.1 years old), of which 10 (15%) had a pericardial effusion [21]. They did not describe the size of the pericardial effusions nor the presence of tamponade physiology [21]. In our study, >10% of patients had a pericardial effusion, albeit insufficient to cause hemodynamic compromise. The small proportion of patients with pericardial effusion is likely due to partial AN treatment in many of our patients. Docx et al. proposed that a pericardial effusion is likely a surrogate marker for disease severity in this patient population [20]. AN results in a decrease in pericardial fat and myocardial muscle wasting, resulting in a pericardial space that is relatively too large for the heart, which is then filled by pericardial fluid. Other proposed mechanisms are associated hypothyroidism and decreased albumin and protein levels caused by starvation [4,8].

Hemodynamic remodeling

Cardiac output (CO) has previously been assessed by echocardiographic studies in the AN population [2,22]. Goldberg et al. studied 14 female patients (18 ± 4 years) with AN [22]. The study demonstrated that the CI was decreased: in controls, the CI was 4.5 ± 1.0 L/min/m^2 ^versus 2.6 ± 0.6 L/min/m^2 ^in AN [22]. Additionally, Olivares et al. evaluated 40 female AN patients (15.5 ± 3.2 years) demonstrating a low CO of 2.9 ± 0.3 L/min at baseline that improved to 3.6 ± 0.3L/min after 9-18 months of treatment [2]. The accuracy of CMR assessment of CO is well known [23-24]. To our knowledge, our study is the first to non-invasively determine CO by CMR in patients with AN. Our CMR-based assessment of CI of 2.8 L/min/m^2^ was low, similar to those published in the literature using echocardiographic-based assessments of CO [2,22].

Structural changes

Structural changes including LV dilation, decreased ejection fraction, and specifically, a decrease in overall LV mass have been well documented using echocardiography and in a single CMR study [2,6-7,11]. In our study, although there were no valvular nor diastolic abnormalities detected, some of the previously known cardiac AN findings such as a decreased LV mass index and smaller chamber sizes were observed.

Previously, Silvetti et al. demonstrated that a low BMI in AN patients correlated with smaller LV chamber measurements [25]. In their study, a total of 23 adolescent females aged 15 ± 2 years with a low BMI of less than 19 with low caloric intake was associated with a smaller IVS thickness in 52% of patients, smaller LV wall thickness in 61% of patients, smaller LA diameter in 31% of patients, and smaller LV mass in 61% of patients [25]. Similar to the study by Silvetti et al., our study demonstrated a significantly lower LV mass index and posterior wall thickness in AN patients of a similar age cohort [25]. 

There has only been a single study to date which utilized CMR in the evaluation of a group of adult patients with AN. In this cross-sectional observational study by Oflaz et al., a total of 68 female patients, aged 22 ± 7 years, were studied including 40 AN patients (BMI: 20 ± 1.6 kg/m^2^) and 28 healthy controls (BMI: 15 ± 1.8 kg/m^2^) [11]. As compared to controls, individuals with AN had a lower LV mass index, smaller IVS, and PWT, and preserved LVEF [11]. Our study results confirm these findings, as we also demonstrated a lower LV mass index, smaller IVS, and smaller PWT.

Myocardial fibrosis

AN patients are at an risk of increased SCD [8,26]. Amongst the potential mechanisms for SCD in this patient population, myocardial fibrosis has been reported in necropsy findings of AN patients [26]. In the study by Oflaz et al., approximately 23% of the patient population demonstrated LGE on CMR imaging indicative of myocardial fibrosis [11]. The presence of LGE of the LV myocardium may be a surrogate marker of an increased risk of SCD, similar to the patient populations with underlying cardiomyopathy and ischemic heart disease [27-29]. As compared to the study by Oflaz et al., we found no evidence of LGE in our patient population [11]. First, our patients were mostly weight recovered and it is not clear that this was the case in the Oflaz study [11]. Second, we question whether there may be different patterns in the use of medications to promote unhealthy weight loss between our population and that of the Oflaz study [11]. Although it is known that AN patients are at higher risk of abusing laxatives, appetite suppressants, and diuretics, neither study specifically evaluated the use of these medications [30]. Further research is required to confirm whether the presence of LGE in AN patients serves as a noninvasive marker of increased SCD in this patient population. 

Limitations

Our study has several limitations. First, this study has a small sample size. As a pilot study, we sought to evaluate for major structural or functional abnormalities, to compare TTE and CMR findings, and to better quantify RV parameters in AN patients. Without knowing the incidence or degree of abnormality, we could not determine in advance the sample size needed to adequately power the study. We recognize that the assessment of RV mass is limited by variability and conclusions regarding differences in RV mass must be made with caution. With regard to LGE, we were unable to perform T1 mapping which may have uncovered more mild degrees of fibrosis. Also, since many of our patients were substantially weight recovered when studied, we may have failed to detect findings that were present at worse levels of starvation. Specifically, the time period between the diagnosis of AN and TTE/CMR imaging was not constant between each patient. Finally, as we did not have an age-matched population of subjects without cardiac disease, we used adult female published literature normal values as a comparator.

## Conclusions

Patients with AN are at risk of developing cardiac complications including heart rate and repolarization abnormalities, pericardial changes, hemodynamic changes, and structural changes. As evidenced by CMR, young patients with AN have lower cardiac mass and volumes with no evidence of myocardial fibrosis. Further research is required to evaluate the role of cardiovascular remodeling in AN patients and the potential role of LGE as a marker of increased SCD.
